# Tensor Product Model Transformation Based Adaptive Integral-Sliding Mode Controller: Equivalent Control Method

**DOI:** 10.1155/2013/726963

**Published:** 2013-12-25

**Authors:** Guoliang Zhao, Kaibiao Sun, Hongxing Li

**Affiliations:** ^1^Faculty of Electronic Information and Electronic Engineering, Dalian University of Technology, Dalian 116024, China; ^2^Modern Manufacture Engineering Center, Heilongjiang University of Science and Technology, Harbin 150022, China

## Abstract

This paper proposes new methodologies for the design of adaptive integral-sliding mode control. A tensor product model transformation based adaptive integral-sliding mode control law with respect to uncertainties and perturbations is studied, while upper bounds on the perturbations and uncertainties are assumed to be unknown. The advantage of proposed controllers consists in having a dynamical adaptive control gain to establish a sliding mode right at the beginning of the process. Gain dynamics ensure a reasonable adaptive gain with respect to the uncertainties. Finally, efficacy of the proposed controller is verified by simulations on an uncertain nonlinear system model.

## 1. Introduction

Sliding mode control (SMC) has advantages like ease of implementation and reduction in the order of the state equation. It also has the ability to withstand external disturbances and model uncertainties satisfying the matching condition, that is, the perturbations that enter the state equation at the same point as the control input. SMC design comprises two steps: a sliding surface is first constructed such that the system trajectories along the sliding surface meet the specified performance and then a discontinuous or continuous control law drives the states towards the sliding surface, and keeps them on the surface thereafter, regardless of disturbances or parasitic uncertainties. The resulting controller, although robust against matched perturbations, still suffers from reaching phase problem, that is, an initial period of time in which the system has not yet reached the sliding surface and it is sensitive to perturbations satisfying the matching condition.

In order to solve the reaching phase problem, an integral-sliding surface was proposed [1, 2]. Successively, many application examples of integral-sliding mode control have been developed in the literature and different systems are studied, such as multi-input multi-output (MIMO) linear system [3], uncertain nonlinear system [4, 5], uncertain stochastic system [6], and uncertain fractional order nonlinear system [7]. Some practical applications are also investigated [8–10]. The basic idea of integral-sliding surface control design is to define the nominal control law, which is responsible for the performance of the nominal system, that is, without perturbations, and a discontinuous or continuous control law, which is responsible for suppression of the parasitic uncertainties and external disturbances or perturbations. In integral-sliding surface control, an integral term is included in the sliding surface, and it allows to define the sliding surface in such a way that the system trajectories start in the sliding motion right at the beginning of the process. Moreover, it is well known that terminal sliding mode control (TSMC) guarantees a finite convergence time, by combining derivative and integral terminal sliding modes in a recursive structure; derivative-integral terminal sliding mode control (DI-TMSC) methods are proposed to achieve exact or approximate finite-time convergence for the output tracking of higher order nonlinear systems [11]. More recently, several works on the TP model transformation based sliding mode control appeared in the literature [12–16].

TP model transformation was first introduced by Baranyi et al. [17, 18]. It allows transforming dynamic system models defined over bounded domains into convex parameter-varying weighted combination of linear time invariant (LTI) systems. This transformation can transform a function into TP function form if it is feasible. If an exact transformation is impossible to achieve, then the method will determine a TP function which can be taken as an approximation. This feature offers the TP model transformation the ability to provide a tradeoff between approximation accuracy and complexity of the resulting TP model. linear parameter varying (LPV) based control has developed based on TP model transformation and linear matrix inequality (LMI) [17–22]. TP model transformation has found many applications in new direction in control theory [16, 23–25]. For further information., please refer to recently published monograph for qLPV control theories [26] and papers [19, 27].

Inspired by the adaptive sliding mode controller [28], the TP model transformation based parallel distributed compensation (TPDC) technique is applied, and a TP model transformation based adaptive integral-sliding mode controller (TPAISMC) is proposed in this paper. By combining with *σ*-adaptation strategy [29], a real sliding surface is realized; due to uncertainties and external disturbances, the phenomenon that adaptive gain is increasing all the time when ideal sliding mode does not occur is avoided. Chattering caused by perturbations and uncertainties [30] is suppressed by TPAISMC with *σ*-adaptation. Furthermore, the design process can be numerically realized without analytical derivation due to the characteristics of the TP model transformation based parallel distributed compensation. Thus, this control scheme has potential applications in many practical environments.

The rest of the paper is organized as follows. In Section 2, the concept of TP model transformation is introduced, and the control problem is formulated. In Section 3, TP model transformation based adaptive integral-sliding model control is presented, and TP model transformation based adaptive integral-sliding model control with *σ*-adaptation method is proposed. In Section 4, the simulations on TP model transformation based adaptive integral-sliding model controllers with different adaptive gain dynamics are carried out, and the control performance is tested. Finally in Section 5 the conclusions are given.

## 2. Preliminaries

### 2.1. Tensor Product Model Transformation

It is known that a given bounded continuous function *g*(*x*) (*x* ∈ ℝ^*N*^) can be approximated by tensor product over a compact domain *Ω*. First, divide the compact domain into *N*-dimensional hyper rectangular grid based on a given grid size; on each dimension, the locations are defined by the vector

(1) $$\begin{array}{c} a_{n}=\left[a_{n,1},a_{n,2},\ldots ,a_{n,M_{n}}\right],\;n=1,2,\ldots ,N, \end{array}$$

where *a*_*n*,1_ ≤ *a*_*n*,2_ ≤ ⋯≤*a*_*n*,*M*_*n*__ and the *N*-dimensional hyper rectangular grid belongs to a bounded region *Ω* = [*a*_1,1_, *a*_1,*M*_1__]×[*a*_2,1_, *a*_2,*M*_2__]×⋯×[*a*_*N*,1_, *a*_*N*,*M*_*N*__]. Then function *g*(*x*) can be sampled over the grid points as

(2) $$\begin{array}{c} g\left(x\right)=b_{i_{1},i_{2},\ldots ,i_{N}}, \end{array}$$

where *x* = [*a*_1,*i*_1__, *a*_2,*i*_2__,…, *a*_*N*,*i*_*N*__] (for all *n*: *i*_*n*_ = 1,2,…, *M*_*n*_). Sampled matrices are stored into a tensor *𝒮* ^*s*^ where superscript “*s*” means “sampled.” Then higher order singular value decomposition (HOSVD) is used to root out the redundant content of the sampled set. It follows that

(3) g(x)≈g^(x)=ℬ⊗n=1Nwn(xn)=∑i1=1I1 ‍∑i2=1I2⋯∑iN=1IN ∏n=1Nwn,in(xn)bi1,i2,…,iN,  

where tensor *ℬ* ∈ ℝ^*I*_1_×*I*_2_×⋯×*I*_*N*_^ and ℝ^*I*_1_×*I*_2_×⋯×*I*_*N*_^ is the vector space of real-valued *I*_1_ × *I*_2_ × ⋯×*I*_*N*_ tensors; *I*_*n*_ is the number of univariate basis functions used in the *n*th dimension of the parameter vector and *b*_*i*_1_,*i*_2_,…,*i*_*N*__ is the entry of *ℬ*. Tensor *ℬ* satisfies the following properties.

(i) All-orthogonality: 〈*ℬ*_*i*_*n*_=*α*_, *ℬ*_*i*_*n*_=*β*_〉 = 0 for all possible values of *α*, *β*, and *n* (*β* ≠ *α*).
(ii) Ordering: ||*ℬ*_*i*_*n*_=1_|| ≥ ||*ℬ*_*i*_*n*_=2_|| ≥ ⋯≥||*ℬ*_*i*_*n*_=*I*_*n*__|| ≥ 0 for all possible values of *n*, where ||·|| is the Frobenius norm. Row vector *w*_*n*_(*x*_*n*_) ∈ ℝ^*I*_*n*_^ contains basis functions, and *w*_*n*_(*x*_*n*_) = [*w*_*n*,1_(*x*_*n*_), *w*_*n*,2_(*x*_*n*_),…, *w*_*n*,*I*_*n*__(*x*_*n*_)] is normalized to one, where *w*_*n*,*i*_(*x*_*n*_)∈[0,1] (*n* = 1,…, *N*; *i* = 1,…, *I*_*N*_). For the LPV state space model, TP model representation can be obtained as
  (4) $$\begin{array}{c} \dot{x}=\overset{r}{\underset{i=1}{\sum }}w_{i}S_{i}\left(p\right)\left(\begin{array}{c} x\left(t\right)\\ u\left(t\right) \end{array}\right)=\overset{r}{\underset{i=1}{\sum }}w_{i}\left(A_{i}x+B_{i}u\right), \end{array}$$

where *S*_*i*_ are the convex hull of the LTI vertex systems and *r* = ∏_*j*=1_^*N*^ *I*_*j*_ is the linear index equivalent of *N*-dimensional array's index *i*_1_, *i*_2_,…, *i*_*N*_. Note that if all the discarded singular values are zero, this is the exact form of the original tensor and the approximation error is zero. If nonzero singular values are discarded, TP approximation error results in the following representation:

(5) $$\begin{array}{c} ||g\left(x\right)-\overset{r}{\underset{i=1}{\sum }}w_{i}\left(A_{i}x+B_{i}u\right)||\leq \delta , \end{array}$$

where *δ* is used to symbolize the approximation error; it is a function of discarded singular values and *w*_*i*_ ∈ [0,1] is the coefficient function.

### 2.2. Problem Statement

Consider a class of *n*th-order uncertain nonlinear systems. The control system is represented in a state-space form as

(6) $$\begin{array}{c} \dot{x}=f\left(x\right)+b\left(x\right)u+w, \end{array}$$

where *x* = [*x*_1_, *x*_2_,…, *x*_*n*_]^*T*^ ∈ ℝ^*n*^ is the state vector, *u* ∈ ℝ is the control input, *f*(*x*) ∈ ℝ^*n*^ and *b*(*x*) ∈ ℝ^*n*^ are the smooth vector fields, and *w* ∈ ℝ^*n*^ is the external disturbance or parasitic uncertainties. Note that (6) is in a controllable form. The system and input matrices *f*(*x*) and *b*(*x*) are not exactly known but assumed to be in the form *f*(*x*) = *f*_0_(*x*) + Δ*f*(*x*) and *b*(*x*) = *b*_0_(*x*) + Δ*b*(*x*), where *f*_0_(*x*) and *b*_0_(*x*) are the nominal parts of *f*(*x*) and *b*(*x*), respectively. Assume that the nominal part *f*_0_(*x*)+ *b*_0_(*x*)*u* of (6) can be transformed into TP models; that is,

(7) $$\begin{array}{c} S\left(p\right)\left(\begin{array}{c} x\left(t\right)\\ u\left(t\right) \end{array}\right)\approx \overset{r}{\underset{i=1}{\sum }}w_{i}\left(A_{i}x+B_{i}u\right)≜A\left(p\left(t\right)\right)x+B\left(p\left(t\right)\right)u. \end{array}$$

Let *E*(*x*, *u*) be the lumped uncertainty and denoted as

(8) $$\begin{array}{c} E\left(x,u\right)=\Delta f\left(x\right)+\Delta b\left(x\right)u+\delta +w. \end{array}$$

Then the uncertain nonlinear system (6) can be represented as

(9) $$\begin{array}{c} \dot{x}=A\left(p\left(t\right)\right)x+B\left(p\left(t\right)\right)u+E\left(x,u\right). \end{array}$$

Assume that the following hypothesis holds for the uncertain nonlinear system (6).

**Assumption 1**

The nominal parts *f*_0_(*x*) and *b*_0_(*x*) of uncertain nonlinear system (6) can be transformed into TP model.

**Assumption 2**

The matrix product *CB*(*p*) is invertible.

**Assumption 3**

The actual values of Δ*f*(*x*), Δ*b*(*x*), and *w* are unknown, but their bound should exist for all *x* and *t*.

**Assumption 4**

The known nominal nonlinear plant of (6), $\dot{x}\left(t\right)=f_{0}\left(x,t\right)+b_{0}\left(x\right)u\left(t\right)$, is globally asymptotically stabilizable via a nominal control *u*_eq_; that is, there is a nonempty set of Lyapunov function *V* such that for any choice of *ℂ* ^1^ function *V*(*x*, *t*) ∈ *V* : ℝ^*n*^ × ℝ^+^ → ℝ^+^ satisfies

(10) $$\begin{array}{c} \gamma_{1}\left(||x||\right)\leq V\left(x,t\right)\leq \gamma_{2}\left(||x||\right),\\ \frac{\partial V}{\partial t}+{\left(\frac{\partial V}{\partial x}\right)}^{T}\left[f\left(x\right)+b\left(x\right)u_{\text{eq}}\right]\leq -\tilde{\gamma }\left(||x||\right), \end{array}$$

where *γ*_1_, *γ*_2_ : ℝ^+^ → ℝ^+^ are class *𝒦*_*∞*_ functions and $\tilde{\gamma }:{\mathbb{R}}^{+}\to {\mathbb{R}}^{+}$ is a class *𝒦* function, where *𝒦* is the set of all continuous functions *α* : [0, *∞*)→[0, *∞*) that are zero at zero, strictly increasing, and continuous and *𝒦*_*∞*_ is the subset of *𝒦* consisting of those functions that are unbounded.

The following nonlinear integral-type sliding surface [31, 32] is used in the adaptive sliding mode control design:

(11) σ(x,t)=Cx(t)−Cx(t0) −C∫t0t[A(p(τ))x+B(p(τ))ueq]dτ=0.

**Remark 1**

The reaching phase is eliminated for nonlinear integral-type sliding surface defined by (11). It is obvious that the sliding mode exists from the very beginning. The additional integral ∫_*t*_0__^*t*^[*A*(*p*)*x* + *B*(*p*)*u*_eq_]*dτ* + *x*(*t*_0_) provides another degree of freedom than the linear sliding surface, and this term can be thought of as a trajectory of the nominal system, that is, the system in the absence of perturbations and in the presence of the nominal control *u*_eq_. In this work, equivalent control *u*_eq_ is given by TP model transformation based parallel distributed compensation (PDC) technique [17]. Moreover, *f*_0_(*x*) is assumed to be in the linear form; that is, *f*_0_(*x*) = *Ax* in [31, 32] is removed. The requirement of *f*_0_(*x*) is just to satisfy Assumption 1 in this paper. Thus, our method can be applied to a variety of nonlinear systems in which TP model transformation can be applied, for example, inverted pendulum system, underactuated robot manipulators [33], translational oscillator with a rotational actuator (TORA) [34], vertical takeoff and landing (VTOL) aircraft [35], and quadrotor helicopter [36].

## 3. TP Model Transformation Based Adaptive Integral-Sliding Mode Control Design

The adaptive sliding mode control law using the integral-sliding surface (11) can be designed as

(12) $$\begin{array}{c} u\left(t\right)=u_{\text{eq}}+u_{\text{as}},\\ u_{\text{eq}}=-\left(\overset{r}{\underset{i=1}{\sum }}w_{i}\left(p\left(t\right)\right)K_{i}\right)x,\\ u_{\text{as}}=-k\left(t\right){\left(CB\left(p\right)\right)}^{-1}\mathrm{sgn}\left(\sigma \left(x,t\right)\right), \end{array}$$

where the gain *k*(*t*) is the adaptive value preserving the sliding mode and sgn⁡(·) is the signum function. Updating law is designed as

(13) $$\begin{array}{c} \dot{k}\left(t\right)=\bar{k}\left|\sigma \left(x,t\right)\right|, \end{array}$$

where $\bar{k}>0$ and *k*(0) > 0. For the nominal feedback, TP model transformation based parallel distributed compensation (TPDC) is used; gains *K*_*i*_ are computed by the following LMI-based stability.

**Theorem 2 (see [21])**

The polytopic model (4) with the equivalent control *u*_*eq*_ (12) is asymptotically stable if there exist *X* > 0 and *M*_*i*_ that satisfy

(14) $$\begin{array}{c} -XA_{i}^{T}-A_{i}X+M_{i}^{T}B_{i}^{T}+B_{i}M_{i}>0, \end{array}$$

for *i* = 1,2,…, *r*, and

(15) −XAiT−AiX−XAsT−AsX  +MsTBiT+BiMs+MiTBsT+BsMi≥0,

for *i* < *s* ≤ *r*, except the pairs (*i*, *s*) such that *w*_*i*_(*p*(*t*))*w*_*s*_(*p*(*t*)) = 0, for all *p*(*t*), and the feedback gains are determined by the solutions *X* and *M*_*i*_ as

(16) $$\begin{array}{c} K_{i}=M_{i}X^{-1}. \end{array}$$

**Theorem (see [21])**

Assume that ||*x*(0)|| ≤ *ϕ*, where *x*(0) is unknown, but the upper bound *ϕ* is known. The constraint ||*u*(*t*)||_2_ ≤ *γ* is enforced at all times *t* ≥ 0 if the following LMIs hold:

(17) $$\begin{array}{c} \phi^{2}I\leq X,\;\;\left(\begin{array}{cc} X & M_{i}^{T}\\ M_{i} & \gamma^{2}I \end{array}\right)\geq 0. \end{array}$$

The aforementioned control algorithm can be summarized as follows.

**Theorem**

Consider the uncertain nonlinear system (6). Given the integral-type sliding surface (11) and the adaptive control law (12), the sliding mode is guaranteed to be reached in finite time.

**Proof**

Assume that there exists a final gain k* such that *u*_as_ = −k*(*CB*(*p*))^−1^sign⁡(*σ*(*x*, *t*)) is the optimal solution for *u*_as_, and k* > |*C*||*E*(*x*, *u*)|. It is always true since adaptive gain *k*(*t*) is increasing. Differentiating the sliding surface (11) with respect to time, it yields

(18) $$\begin{array}{c} \dot{\sigma }=C\dot{x}\left(t\right)-CA\left(p\left(t\right)\right)x-CB\left(p\left(t\right)\right)u_{\text{eq}}+CE\left(x,u\right). \end{array}$$

A Lyapunov function is chosen as $V=\sigma^{2}/2+{\tilde{k}}^{2}/(2\bar{k})$, where $\tilde{k}=k-k^{*}$. Differentiating *V* with respect to time using (12) and (18), it yields

(19) V˙=σσ˙+k~k~˙k¯=σ[Cx˙(t)−CA(p)x−CB(p)ueq]+(k−k∗)k~˙k¯=σ[CE(x,u)+CB(p)(ueq+uas)−CB(p)ueq] +(k−k∗)|σ(x,t)|.

By applying *u*_as_ = −*k*(*t*)(*CB*(*p*))^−1^sgn⁡(*σ*(*x*, *t*)), (19) becomes

(20) $$\begin{array}{c} \dot{V}=\sigma \left|CE\left(x,u\right)\right|-k^{*}\left|\sigma \right|<0. \end{array}$$

It can be inferred that *σ* and $\tilde{k}$ reach zero in finite time; that is, *σ* → 0 and $k-k^{*}=\tilde{k}\to 0\Rightarrow k\to k^{*}$. The convergence of *σ* and $\tilde{k}$ is proven by Lyapunov stability criterion. In practice, the discontinuous switching function sgn⁡(*σ*) in (12) may cause chattering in the sliding mode. In order to alleviate the input chattering, switching function sgn⁡(*σ*) is replaced with the saturation function sat⁡(*σ*/*ψ*), where Φ is the boundary layer width which should be carefully chosen according to each particular control system. This completes the proof.

**Remark 5**

Briefly, the proposed TPAISMC scheme provides the following three main advantages. First, the knowledge of the upper bound of the system uncertainties is not required. Second, TP model transformation has the ability to provide a tradeoff between approximation accuracy and complexity of the resulting TP function. If an exact transformation is impossible, approximation error is bounded by discarded singular values, then this error can be incorporated into lumped uncertainty *E*(*x*, *u*). Third, the tracking performance of the control system is guaranteed.

The above adaptive method does not require knowledge of uncertainties or perturbations bound. Adaptive gain *k*(*t*) may increase all the time when *σ*(*x*, *t*) ≠ 0. However, this happens all the time in real applications due to sampled computation, noisy measurements, or other nonidealities, sliding surface can hardly achieve zero exactly. The method referred to as “*σ*-adaptation” proposed in [29] is adopted to counteract the uncertainties and perturbations effects; based on integral-sliding surface and TP model transformation, the adaptive sliding mode controller with *σ*-adaptation is given as follows:

(21) $$\begin{array}{c} u\left(t\right)=u_{\text{eq}}+u_{\text{as}},\\ u_{\text{eq}}=-\left(\overset{r}{\underset{i=1}{\sum }}w_{i}\left(p\left(t\right)\right)K_{i}\right)x,\\ u_{\text{as}}=-k\left(t\right){\left(CB\left(p\right)\right)}^{-1}\mathrm{sgn}\left(\sigma \left(x,t\right)\right), \end{array}$$

where the gain *k*(*t*) is defined as

(22) k˙(t){k−|σ(x,t)|sgn⁡(|σ(x,t)|−ϵ)if  k(t)>μ,|σ(x,t)|if  k(t)≤μ,

where *μ* is a prescribed positive parameter. By Lemma 2 [29], there exists a positive constant k* such that *k*(*t*) ≤ k* for all *t* > 0 and k* > |*CE*(*x*, *u*)|. Furthermore, (18) can be simplified as

(23) $$\begin{array}{c} \dot{\sigma }=CE\left(x,u\right)+CB\left(p\right)u_{\text{as}} \end{array}$$

by uncertain nonlinear system (6) and *u*(*t*) is given by (21). Choose Lyapunov function as $V=\sigma^{2}/2+{\tilde{k}}^{2}/(2\bar{k})$. Differentiating *V* with respect to time, using (21) and (23), it yields

(24) V˙=σσ˙+k~k~˙k¯≤σ|C||E(x,u)| −k|σ|+(k−k∗)|σ|sgn⁡(|σ|−ϵ),

when *k* > *μ*. Suppose that |*σ* | >*ϵ*, we have

(25) $$\begin{array}{c} \dot{V}\leq \sigma \left|C\right|\left|E\left(x,u\right)\right|-k^{*}\left|\sigma \right|<0. \end{array}$$

Suppose that |*σ* | <*ϵ*, $\dot{V}$ could be sign indefinite, |*σ*| can increase over *ϵ*, then adaptive gain will increase to guarantee the sliding motion, and then the control strategy ensures that |*σ* | <*ϵ* again. When *k* ≤ *μ*, differentiating *V* with respect to time, using (22), it yields

(26) $$\begin{array}{c} \dot{V}\leq \sigma \left|C\right|\left|E\left(x,u\right)\right|-k\left|\sigma \right|+\left(k-k^{*}\right)\left|\sigma \left(x,t\right)\right|<0. \end{array}$$

In the adaptive sliding mode controller design, *σ*-adaptation is used to regulate the adaptive gain, combining with TPPDC, the following theorem holds.

**Theorem**

Consider the uncertain nonlinear system (6). Given the integral-type sliding surface (11) and the control law (21), the sliding mode is guaranteed to be reached in finite time.

## 4. Simulations

Consider the following nonlinear system:

(27) x˙1=x2, x˙2=x3,x˙3=−(1+0.3sint)x12−(1.5+0.2cos⁡t)x2 −(1+0.4sint)x3+(3+cos⁡x1)u+w.

The nominal parts of system are *f*_0_(*x*) = [*x*_2_, *x*_3_, *x*_1_^2^ − 1.5*x*_2_ − *x*_3_]^*T*^ and *b*_0_(*x*) = [0,0, 3]^*T*^. TP model transformation is used to decompose [*f*_0_(*x*), *b*_0_(*x*)], and the LTI systems are given by

(28) [A1B1]=(010000103−1.5−13),[A2B2]=(01000010−3−1.5−13).

**Figure 1:**
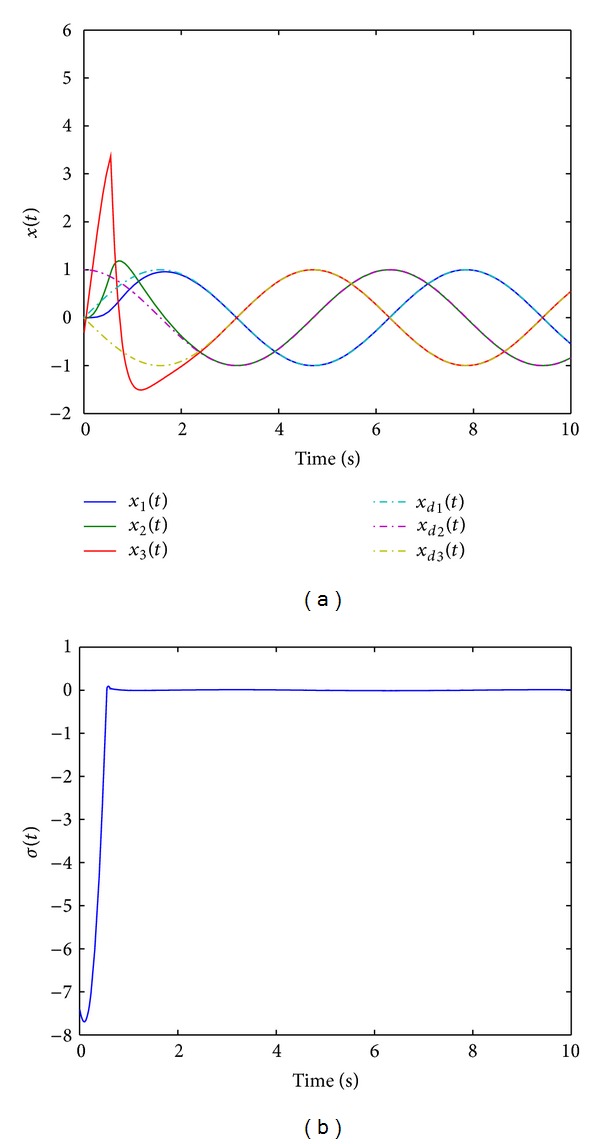
(a) States responses with TPASMC. (b) Sliding surface function with TPASMC.

**Figure 2:**
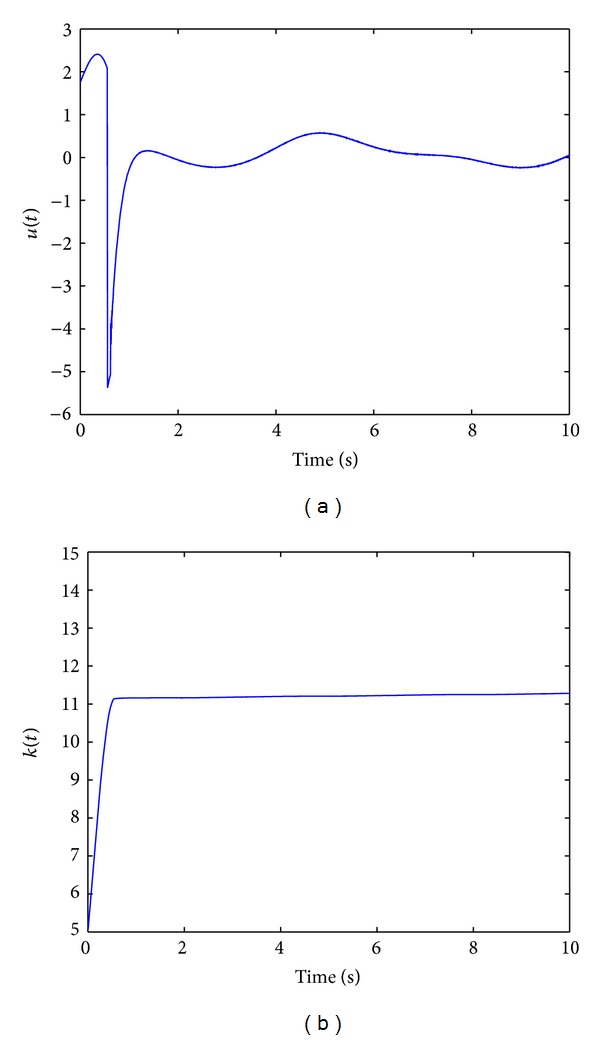
(a) Control input with TPASMC. (b) Adaptive gain parameter.

Since the equivalent control method used in ASMC [28] is replaced with TPDC, the newly proposed method is referred to as TP based adaptive sliding mode control (TPASMC). States responses with TPASMC are shown in Figure 1(a), the sliding surface function is shown in Figure 1(b), and control input and adaptive gain parameter are given in Figures 2(a) and 2(b), respectively. We can conclude that the equivalent control based on TPDC used in TPASMC has the same performance as ASMC which is proposed by Huang et al. [28]. However, since the TPDC is a numerical method, we can design the adaptive sliding mode controller without knowing the analytical system model. There is no modeling error for the nominal system when all the nonzero singular values are used in TP model transformation. The boundary layer technique is applied to avoid high-frequency control activity; the signum function in *u*_as_ is replaced with sat⁡(*σ*/*ψ*), and we have

(29) $$\begin{array}{c} \mathrm{sat}\left(\frac{\sigma }{\psi }\right)=\{\begin{array}{cc} \frac{\sigma }{\psi } & \left|\frac{\sigma }{\psi }\right|\leq 1,\\ \mathrm{sgn}\left(\frac{\sigma }{\psi }\right) & \left|\frac{\sigma }{\psi }\right|>1, \end{array} \end{array}$$

where *ψ* is the boundary layer width. In the simulation, *γ* = 0.5, *ϕ* = 0.1, *C* = [12,7, 1], Γ_0_ = 5, *ψ* = 0.05, the initial state vector for all the simulations is *x*(0) = [0,0, −0.35]^*T*^, and *α* = 0.1 is the decay rate for TPDC. The desired trajectory is supposed to be *x*_*d*_(*t*) = [sin*t*, cos⁡*t*, −sin*t*]^*T*^. The disturbance *w* is supposed to be a random noise possessing a mean value of 0.5 and |*w* | ≤0.1.

**Figure 3:**
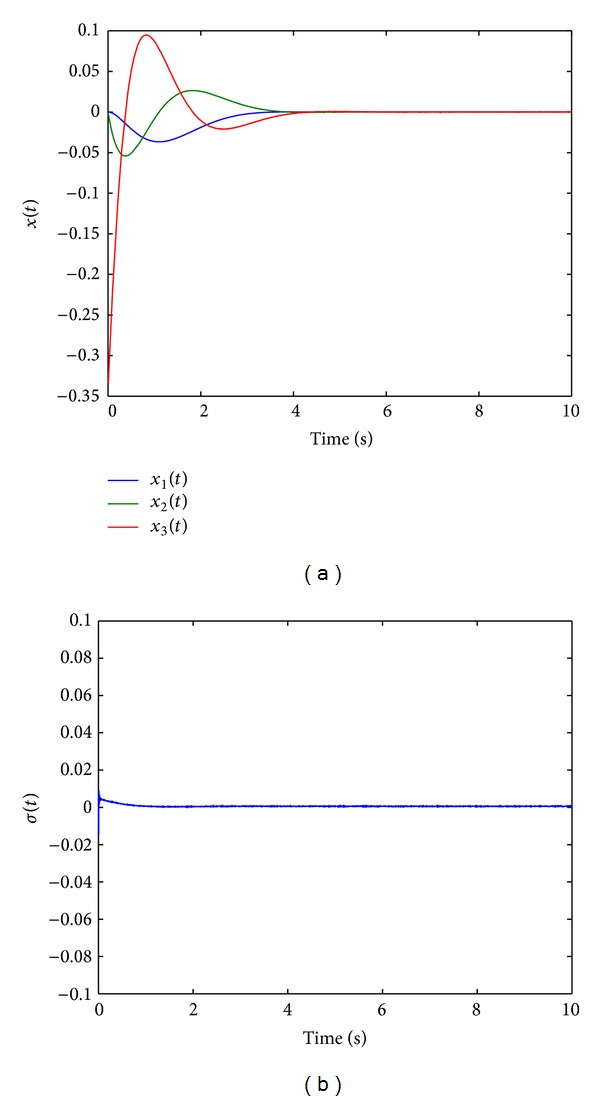
(a) States responses with TPAISMC. (b) Sliding surface function with TPAISMC.

**Figure 4:**
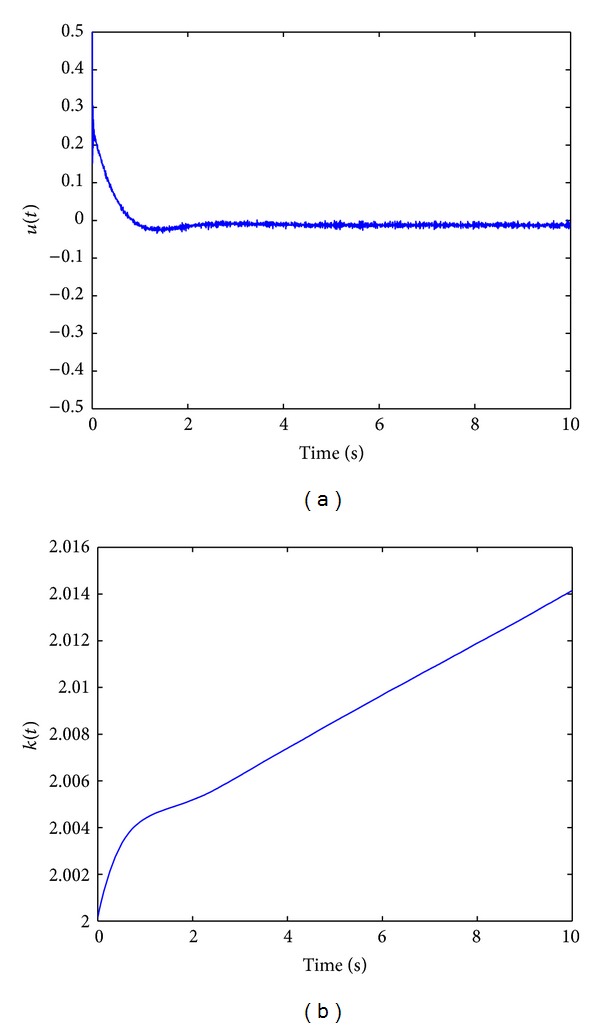
(a) Control input with TPASMC. (b) Adaptive gain parameter.

Figure 3(a) shows the states responses of TPASMC with integral-sliding surface (TPAISMC), sliding surface function is shown in Figure 3(b), and Figures 4(a) and 4(b) show the control input and adaptive gain parameter, respectively. To implement TPAISMC, the following parameters are used:

(30) $$\begin{array}{c} C=\left[5,8,3\right],\;\psi =0.005,\;\Gamma_{0}=2,\\ \gamma =5,\;\;\phi =0.5,\;\;\alpha =1. \end{array}$$

For integral-type sliding surface, reaching phase is eliminated; this can be verified by Figure 3(b). However, in real application, the ideal sliding surface does not exist, then the adaptive gain may increase forever (as shown in Figure 4(b)) when the sliding surface reaches nonzero; the adaptive strategy (13) may cause chattering when the adaptive gain grows large.

To avoid large adaptive gain and chattering, we apply the adaptation law (22) to reduce the chattering. For TPAISMC with *σ* adaptation, the following parameters are used:

(31) $$\begin{array}{c} C=\left[5,8,3\right],\;\;k\left(t_{0}\right)=0.5,\;\;\bar{k}=0.05,\\ \epsilon =0.002,\;\;\mu =0.6,\\ \psi =0.005,\;\;\phi =0.5,\\ \gamma =5,\;\;\alpha =1. \end{array}$$

**Figure 5:**
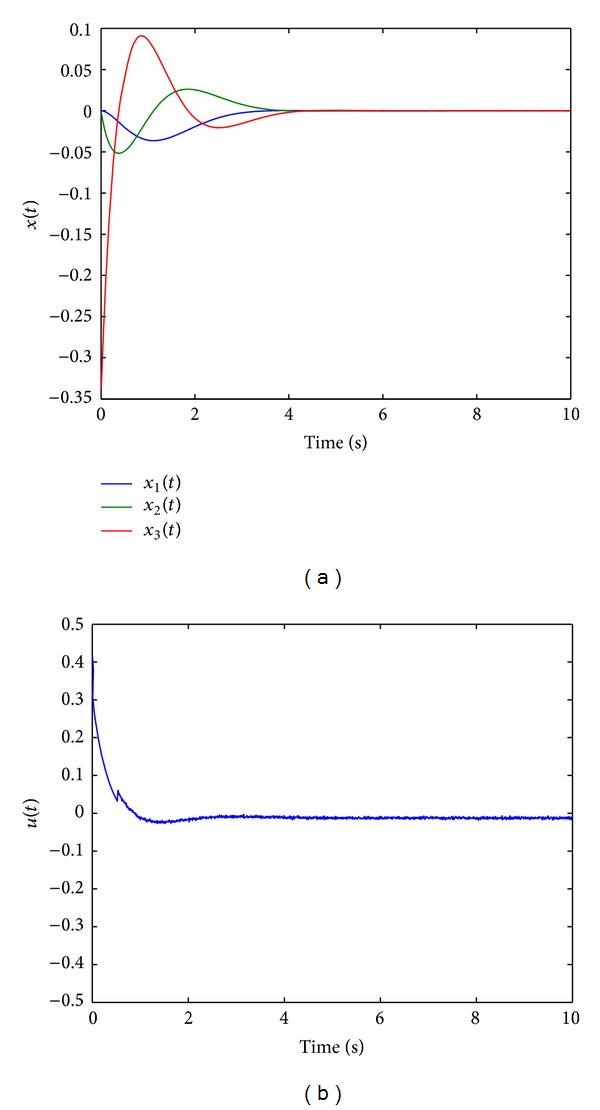
(a) States responses with TPAISMC (*σ*-adaptation). (b) Control input with TPAISMC (*σ*-adaptation).

**Figure 6:**
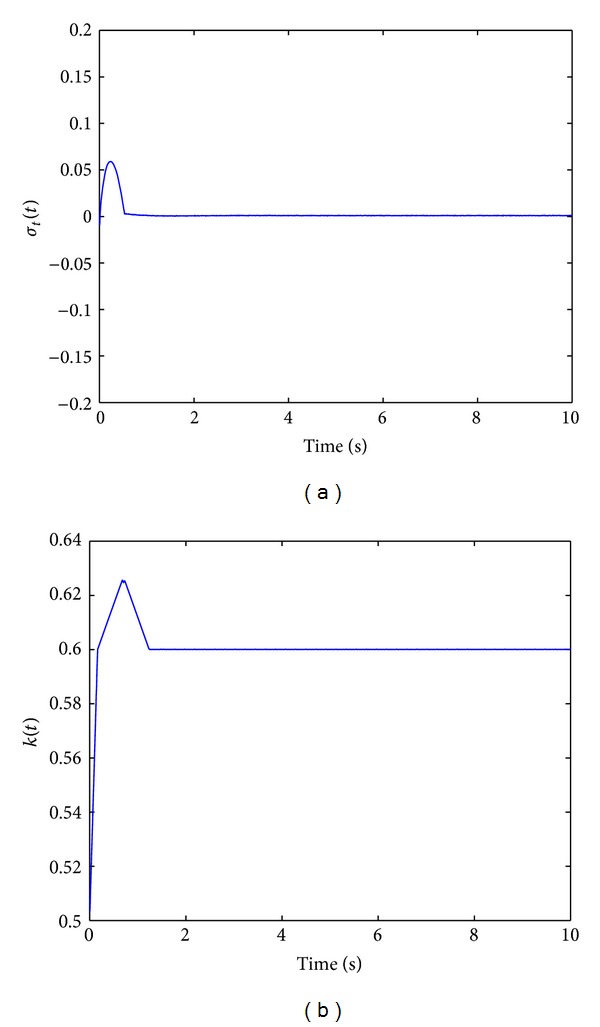
(a) Sliding surface function with TPAISMC (*σ*-adaptation). (b) Adaptive gain parameter (*σ*-adaptation).

Figure 5(a) shows the states responses with TPAISMC when *σ*-adaptation is used. Sliding surface function is shown in Figure 5(b) and control input and adaptive gain parameter are shown in Figures 6(a) and 6(b), respectively. Here it is clearly seen from Figure 5(b) that TPAISMC with *σ*-adaptation is effective in chattering suppression. Adaptive gain *k*(*t*), defining the chattering amplitude in the sliding mode, increases to a reasonable level to guarantee a real sliding mode.

## 5. Conclusion

In this work, an adaptive siding mode controller scheme for a class of uncertain nonlinear system is studied, and an integral-sliding surface was adopted in the designing of TPAISMC. Instead of using the traditional feedback control, TP model transformation based parallel distributed compensation controller was applied to stabilize the states. It is noted that the modeling error caused by TP model transformation can be zero if nonzero singular value is discarded. Nonzero modeling error can be modeled as lumped uncertainties, combining modeling error with perturbations and external disturbances, and can be suppressed effectively by adaptive gain controller with *σ*-adaptation strategy.
